# Protocol for monitoring mRNA translation and degradation in human cell-free lysates

**DOI:** 10.1016/j.xpro.2025.104073

**Published:** 2025-09-10

**Authors:** Nino Schwaller, Evangelos D. Karousis

**Affiliations:** 1 Department of Chemistry, Biochemistry and Pharmaceutical Sciences, University of Bern, Bern, Switzerland; 2 Multidisciplinary Center for Infectious Diseases, University of Bern, Bern, Switzerland

**Keywords:** cell Biology, cell culture, Molecular Biology, gene Expression, Molecular/Chemical probes, Biotechnology and bioengineering

## Abstract

Cell-free translation is a powerful tool for studying protein synthesis. Here, we present a protocol for monitoring mRNA translation and degradation using a human cell-free translation system. The protocol includes instructions for preparing translation-competent lysates via dual centrifugation, generating and capping reporter mRNAs compatible with luciferase assays, performing cell-free translation reactions, and assessing mRNA stability by northern blotting. The protocol supports comparative analysis of translation efficiency and mRNA decay for various 5′ UTRs and viral RNA elements.

For complete details on the use and execution of this protocol, please refer to Gurzeler et al.^1^ and Bäumlin et al.^2^

## Highlights

• Instructions for monitoring RNA fate and translation efficiency in human lysates
• Steps for decoupling and distinguishing translation inhibition from mRNA degradation
• Adaptable steps to test small molecules or viral/cellular translation regulators
• Compatibility guidance for a wide range of downstream methods

## Abstract (editor highlights)

Publisher’s note: Undertaking any experimental protocol requires adherence to local institutional guidelines for laboratory safety and ethics.

## Graphical abstract

**Figure:**
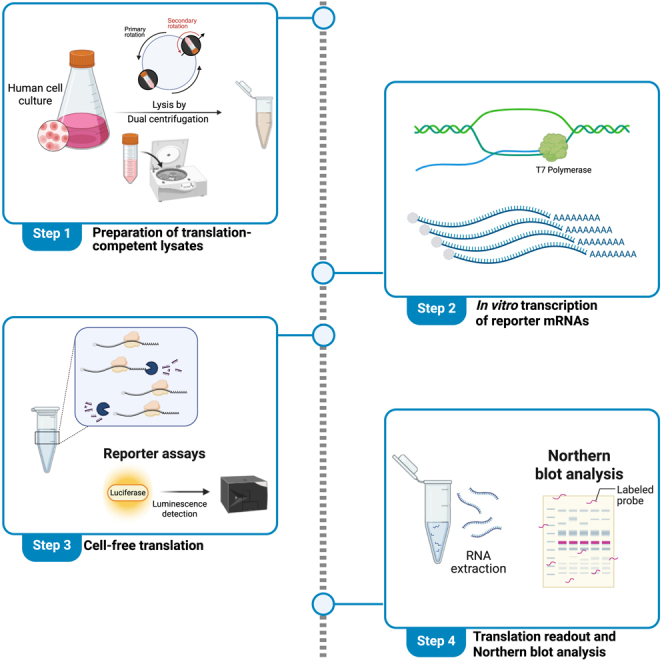

## Before you begin

This protocol is designed to generate translation-competent lysates that will allow researchers to monitor and uncouple the processes of human mRNA translation and degradation. In addition, instructions on the *in vitro* transcription of reporter mRNA containing an efficient 5′ UTR, a *Renilla* luciferase (Rluc) open reading frame (ORF) and a template-encoded poly(A) tail are also provided. While this protocol is optimized for HeLa S3 cell lysates prepared by dual centrifugation and Rluc reporter mRNAs, it is compatible with a range of *in vitro* transcribed RNAs and can be modified to yield lysates from different cell lines and address the impact of recombinant proteins on mRNA translation, or degradation.^2^^,^^3^^,^^4^ Ensure all buffers and reagents are freshly prepared and thawed on ice. Maintain RNase-free conditions throughout the protocol.

### Innovation

This protocol describes the preparation and use of large quantities of translation-competent human cell-free lysates that enable simultaneous measurement of mRNA translation and degradation within the same experimental setup. The lysates are generated using a dual-centrifugation approach that tightly controls mechanical disruption force and temperature, ensuring high reproducibility between preparations and preserving the integrity of translation and RNA decay machineries.

Because these extracts retain intact ribosomes, translation factors, and RNA turnover components, they provide a reaction environment that closely reflects the physiological conditions of the source cell line while eliminating upstream cellular signaling complexity. The system is compatible with a wide range of RNA reporters, capped or uncapped, polyadenylated or not, and of viral or host origin, and supports systematic investigation of sequence or structural elements such as 5′ untranslated regions, internal ribosome entry sites (IRESs), and regulatory RNA–protein interactions.

The protocol’s scalability allows the preparation of lysates in large batches, enabling consistent experimental conditions across multiple assays and reducing batch-to-batch variability. Reactions can be precisely manipulated by adding purified proteins, inhibitors, or viral components, facilitating mechanistic studies of translation regulation and mRNA stability. This streamlined approach eliminates the need for separate experimental systems to monitor translation and decay, reduces hands-on time, and increases throughput.

### Institutional permissions

This study uses commercially available human-derived cell lines. All biosafety procedures must comply with institutional guidelines. This protocol involves the use of radioactive isotopes (e.g., α-^32^P-dCTP). All handling, storage, and disposal of radioactive materials must comply with institutional radiation safety regulations and be approved by the appropriate institutional biosafety or radiation protection office.

### HeLa S3 cell culture for lysate preparation

**Timing: 5–7 days before lysate preparation**

This procedure describes the expansion and transition of HeLa S3 cells from adherent to suspension culture before lysate preparation.

1. Thawing and Recovery.
  a. Thaw a frozen HeLa S3 vial containing 5 × 10^6^ cells in a 37°C water bath and transfer to 10 mL of pre-warmed DMEM medium supplemented with FCS and antibiotics (DMEM+/+).
  b. Pellet cells at 200 × g for 5 min, discard the supernatant, and resuspend in 10 ml fresh DMEM+/+.
  c. Seed into a 75 cm^2^ tissue culture flask and grow at 37°C, 5% CO_2_ until ∼80%–90% confluency.
2. Adherent Expansion.
  a. Passage the cells twice in adherent format using trypsinization. Switch to a 150 cm^2^ tissue culture flask after the first passage to increase the cell yield.
  b. Monitor viability using Trypan Blue; ensure >90% viability before transitioning to suspension.
3. Transition to Suspension Culture.
  a. Transfer cells into sterile, vented suspension flasks in DMEM +/+.
  b. Start with approximately 20 mL and gradually add DMEM +/+ to maintain a cell density between 0.15 × 10^6^ and 1.2 × 10^6^ cells/mL. Increase the culture volume and switch to larger flasks as needed to avoid exceeding one-third of the flask’s total volume.
  c. Incubate at 37°C in a humidified shaker incubator at 80–100 rpm and 5% CO_2_.

***Note:*** We normally add fresh DMEM +/+ every other day, diluting the cells 1:4 to maintain optimal density based on the ∼24-h doubling time of HeLa S3 cells.

**CRITICAL:** For optimal consistency and translational activity, HeLa S3 cells should not be maintained in continuous culture for more than 4 weeks. Extended culture increases the risk of genetic drift, reduced lysate quality, and contamination. For high reproducibility, we recommend preparing fresh cultures from early-passage frozen stocks for each batch of lysate preparation.

## Key resources table

**Table:** 

REAGENT or RESOURCE	SOURCE	IDENTIFIER
**Biological samples**		

HeLa S3 lysate	Custom preparation	Prepared as described in the Star Protocol

**Chemicals, peptides, and recombinant proteins**		

[α-^32^P]dCTP	Hartmann Analytic	Cat. No. SCP-205
Citrate buffer 1 mM, pH 6.5	Invitrogen	Cat. No. AM7001
CleanCap reagent AG	TriLink BioTechnologies	Cat. No. N-7113-5
CloneAmp HiFi premix	Takara	Cat. No. 639298
Creatine kinase	Roche	Cat. No. 10127566001
Creatine phosphate	Roche	Cat. No. 10621714001
CutSmart buffer 10×	New England Biolabs	Cat. No. B6004S
Cycloheximide	Fluorochem	CAS. No. 66-81-9 Cat. No. M01527
DL-dithiothreitol (DTT) 1 M in H_2_O	Merck	Cat. No. 43816-10ml
DMEM	Gibco	Cat. No. 41966-029
EDTA	Sigma-Aldrich	Cat. No. E5134-500G
Fetal calf serum	BioConcept	Cat. No. 2-01F30-I
Formaldehyde loading dye (NorthernMax)	Invitrogen	Cat. No. AM8552
Gel loading dye, purple (6×)	New England Biolabs	Cat. No. B7024S
HEPES 1 M, pH 7.3	Fisher Bioreagents	Cat. No. BP299-100
*Hind*III-HF	New England Biolabs	Cat. No. R3104
Inorganic pyrophosphatase (PPase) (0.1 U/μL)	Thermo Scientific	REF: EF0221
K-acetate 3 M, pH 5.3	Jena Bioscience	Cat. No. BU-107
KCl 1 M	Thermo scientific	Cat. No. J62422.AK
LDS sample loading buffer 4×	Invitrogen	Cat. No. NP0008
MES	Sigma	Cat. No. M3671-250G
MgCl_2_ 1 M	Jena Bioscience	Cat. No. BU-110-1M
Murine RNase inhibitor	Vazyme	Cat. No. R301-01-AA
NaCl 5 M in H_2_O	Sigma	Cat. No. S5150-1L
Phosphate-buffered saline (PBS), 1×, pH 7.4	Gibco	Cat. No. 10010-031
Protease inhibitor cocktail 100×	Bimake	Cat. No. B14002
Puromycin	Santa Cruz Biotechnology	Cat. No. sc-108071
rATP (100 mM)	Thermo Scientific	REF: R0441
rCTP (100 mM)	Thermo Scientific	REF: R0451
rGTP (100 mM)	Thermo Scientific	REF: R0461
rUTP (100 mM)	Thermo Scientific	REF: R0471
RNase-free water	Sigma	Cat. No. W4502-1L
SDS 10%	Fisher Bioreagents	Cat. No. BP2436-1
T7 RNA polymerase	Thermo Scientific	REF: EP0111
T7 transcription buffer 5×	Thermo Scientific	REF: EP0111
Tris base	Fisher Bioreagents	Cat. No. BP152.5
Tris-HCl pH 7.5	Fisher Bioreagent	Cat. No. BP1756-100
Trypan blue stain	Gibco	Cat. No. 15250-061
Trypsin-EDTA PBS	BioConcept	Cat. No. 5-51F00-H
Turbo DNase (2 U/μL)	Thermo Scientific	REF: AM2238
ULTRAhyb ultrasensitive hybridization buffer	Invitrogen	Cat. No. AM8669

**Critical commercial assays**		

Renilla-Glo luciferase assay system	Promega	Cat. No. E2720
Maxwell RSC simplyRNA cells kit	Promega	Cat. No. AS1390
Monarch RNA cleanup kit	New England Biolabs	Cat. No. T2040L
ChIP DNA Clean & Concentrator kit	Zymo Research	Cat. No. D5205
Vaccinia capping system	New England Biolabs	Cat. No. M2080S
DecaLabel DNA labeling kit	Thermo Scientific	Cat. No. K0622
Amersham MicroSpin G-50 columns	Cytiva	Cat. No. 27533001

**Experimental models: Cell lines**		

HeLa S3 cells	ATCC	Cat. No. CCL-2.2

**Oligonucleotides**		

p200 5′UTR hRLuc for northern blotting probe preparation	5′-TCTGCAGAATTCGCCCTTCATG-3’ 5′-GCACGTTCATTTGCTTGCA-3′	

**Recombinant DNA**		

p200: pCRII-RLuc-SV40-6xMS2-A80	Described in Karousis et al.^5^	N/A

**Software and algorithms**		

GraphPad Prism	GraphPad Software	Version 10.0.2 https://www.graphpad.com/updates/prism-1002-release-notes
ImageJ	NIH	Version 1.52 https://imagej.net/ij/download.html
Excel	Microsoft	Version 2403 https://www.microsoft.com/en-us/microsoft-365/download-office

**Other**		

2 L shaker flask – Nalgene Erlenmeyer PP	Nalgene	Cat. No. 4102-2000
250 mL shaker flask – Erlenmeyer PC, non-treated	Avantor	Cat. No. 214-0449
125 mL shaker flask – Erlenmeyer PC	Corning	Cat. No. 430421
75 cm^2^ or 150 cm^2^ cell culture flask, surface treated	TPP	Cat. No. 90076 or 90151
Dual centrifuge ZentriMix 380 R	Hettich AG	N/A
2 mL screw cap microtubes for dual centrifuge	Sarstedt	Cat. No. 72.693
White-bottom 96-well plate	Greiner	Cat. No. 655073
Microplate reader	Promega GloMax Explorer or similar	GM3500
UV irradiation chamber for crosslinking	Stratalinker or similar	N/A
Nylon transfer membrane	Cytiva, Amersham	RPN303B

## Materials and equipment

**Table:** Translation Buffer (TB) for lysate preparation

Reagent stock	Final concentration	Amount (μL)
HEPES 1M, pH 7.3	33.78 mM	33.8
K-acetate 3M, pH 5.3	63.06 mM	21.0
MgCl_2_ 25 mM	0.68 mM	27.0
KCl 1M	54.05 mM	54.1
Creatine phosphate (500 mM)	13.51 mM	27.0
Creatine kinase (5 μg/ml)	230 ng/ml	45.9
Protease inhibitor cocktail 100×	1×	10.0
MilliQ water	to 1000 μL	781.1
**Total**	–	**1000 = 1 mL**

Store at 4°C for not more than 6 h.

## Step-by-step method details

### Preparation of HeLa S3 lysates

**Timing: approximately 2 h**

This step yields translation-competent lysates that can resist multiple freeze-thaw cycles.^1^

1. Before starting, pre-cool the PBS and translation buffer (TB), the centrifuges needed for cell culture pelleting, dual centrifugation and post-lysis centrifugation.

***Note:*** Pre-cooling of the dual centrifuge device can be performed at 500 rpm for 15 min at −5°C.

2. Grow HeLa S3 cells to ∼0.8–1.5 × 10^6^ cells/mL with >90% viability.

***Note:*** We typically use culture volumes of 250 mL to 1 L, which yields 1–6 mL of translation-competent lysate.

**Caution:** Cell viability >90% is essential for yielding highly translation-competent lysates.

**CRITICAL:** If you prepare lysates from adherent cells, do not detach them by scraping, as it destroys the translation potency of the produced lysates.^6^ Always detach with trypsinization.

3. Perform cell counting by isolating a cell culture sample (2-4 mL), 5 sec vortex to reduce cell clumps.

**CRITICAL:** Accurate cell counting is essential for yielding highly translation-competent lysates. Perform multiple counts if cell numbers are inconsistent, or dilute the cells to avoid cell clumps.

4. Based on the living cell counts, calculate the volume of TB needed for lysate preparation, using 2 × 10^8^ cells/ml TB.

***Note:*** Prepare the translation buffer shortly before usage and keep it on ice.

5. Based on the calculations of step 4, prepare the required volume of TB (see materials and equipment setup section of this method) and keep on ice.
6. Pellet the cells by centrifugation at 500 × *g* for 5 min at 4°C, in 50 mL or 500 mL centrifuge tubes, depending on the scale. Discard the supernatant.

***Note:*** We recommend using a vacuum pump to remove supernatant as the cell pellet may be unstable.

**CRITICAL:** All following steps should be performed using ice-cold buffers and cells should be continuously kept on ice. Proceed to the next step as soon as possible within ≤10–15 min (do not exceed 20 min) to preserve lysate translational activity.

7. Wash I: Tap gently on the tube to loosen the pellet and resuspend the cell pellet in 20 mL ice-cold PBS and transfer cells into a 50 ml tube. Spin down at 500 × *g*, 5 min, 4°C and discard the supernatant.

***Note:*** For convenient handling, we recommend resuspending each pellet (from 500 mL of culture) in PBS and transferring it into an individual 50 mL conical tube for the wash step. Wash gently by detaching the pellet, and avoid full resuspension during the washing steps, to prevent lysis due to pipetting.

8. Wash II: Resuspend the cell pellet in 5 mL of ice-cold PBS, keeping it in the same 50 mL tube. Spin down at 500 × *g*, 5 min, 4°C and discard the supernatant.
9. Add the calculated volume of TB buffer to the cell pellets, gently resuspend the cells using a 1 mL pipette, and combine them in one 50 mL tube.

***Note:*** The cell suspension will be highly concentrated. To minimize cell loss, use only a portion (∼4/5) of the total TB buffer to sequentially resuspend each pellet in its original tube, combining them into a single 50 mL tube. After all pellets are combined, use the remaining TB buffer to rinse the original tubes and transfer the rinses to the same 50 mL tube.

10. Distribute the cell suspension in TB into 2 mL dual centrifuge-compatible tubes.

**CRITICAL:** To ensure equal lysis forces during dual centrifugation, the cell suspension must be homogeneous, and the volumes shared equally in dual centrifugation-compatible tubes. We recommend using equal volumes of 400 to a maximum of 800 μL per 2 mL dual centrifugation tube.

***Note:*** Given the high cell concentration, the final volume of the suspension is approximately 1.5 times the volume of the added TB.

11. Perform dual centrifugation for 4 min, 500 rpm, −5°C.

***Note:*** These conditions are optimized for HeLa S3 cells. For guidelines using adherent cell lines and optimizing dual centrifugation conditions, refer to Ziegelmüller et al., 2025.^6^

12. Pellet the cell debris using a regular table-top centrifuge at 13′000 × g for 10 min at 4°C
13. Collect and pool the supernatant, invert gently 4–5 times to mix.

**CRITICAL:** Pooling and gentle mixing are essential to guarantee reproducible translation efficiency within different vials.

14. Prepare aliquots of the lysate, typically 50–200 μL, to avoid repeated freeze-thaw cycles.
15. Snap-freeze the aliquots in liquid nitrogen and store at −70°C.

### *In vitro* transcription and capping of reporter mRNAs

**Timing: 6–8 h**

This procedure describes the generation of capped luciferase reporter mRNAs from plasmid DNA using either co-transcriptional or enzymatic capping strategies. The linearized DNA template preparation and transcription are common for both methods. Co-transcriptional capping is described at the step of *in vitro* transcription. Enzymatic capping is done at a separate step.

**Figure 1:**
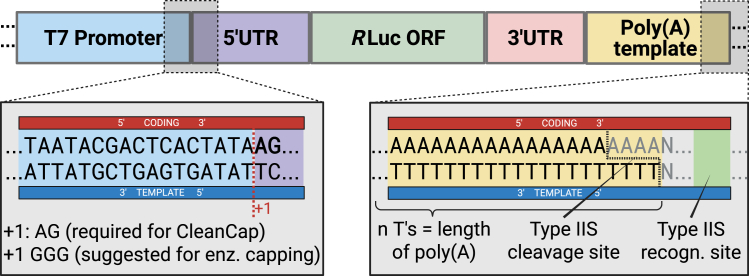
Design and features of reporter mRNA plasmids used for *in vitro* transcription Schematic overview of plasmid elements required for the generation of capped reporter mRNAs by T7 run-off transcription. Reporter plasmids contain a T7 promoter followed by a CleanCap-compatible AG transcription initiator, a synthetic 5′ untranslated region (5′UTR), a Renilla luciferase (RLuc) open reading frame (ORF), a 3′UTR, and a template-encoded poly(A) sequence. The left inset highlights the use of an AG transcription start dinucleotide, required for efficient co-transcriptional capping, with the CleanCap AG reagent. The inset on the right shows the poly(A) template configuration, including a type IIS restriction site downstream of the poly(T) stretch, to enable precise 3′ end definition by enzymatic linearization (e.g., *BsmF*I). This plasmid layout ensures compatibility with cap-dependent translation initiation and enables the controlled generation of reporter mRNAs with defined untranslated regions and polyadenylation sites.

**Figure 2:**
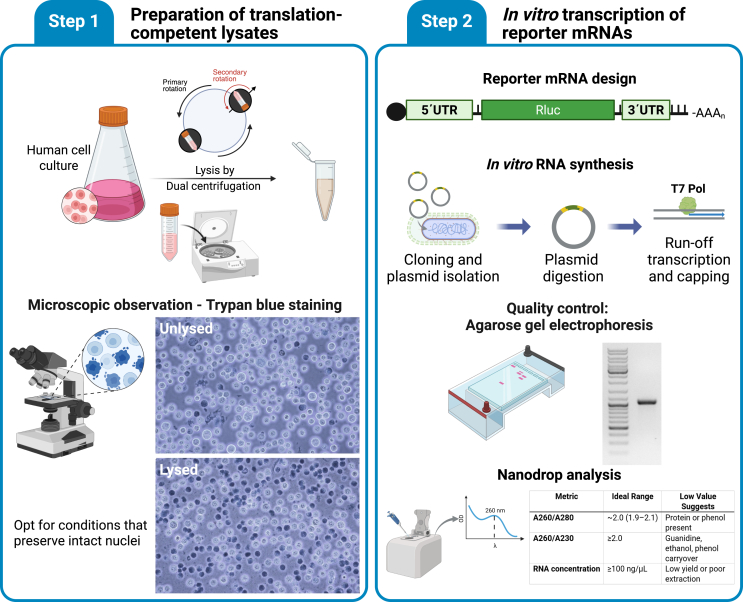
Preparation of translation-competent lysates and *in vitro* transcription of reporter mRNAs (Step 1) Human cell cultures (e.g., HeLa S3) are harvested and subjected to dual centrifugation to generate translation-competent lysates. Lysis is assessed microscopically using Trypan Blue staining. Optimal lysis conditions preserve nuclear integrity, as indicated by intact nuclei post-lysis. (Step 2) Reporter mRNAs are generated via *in vitro* transcription using linearized plasmids containing 5′ and 3′ UTRs flanking an RLuc ORF. After plasmid digestion, run-off transcription and co- or post-transcriptional capping are performed using T7 RNA polymerase. RNA quality is assessed via agarose gel electrophoresis and NanoDrop spectrophotometry. Ideal A_260_/A_280_ and A_260_/A_230_ ratios indicate low contamination, while concentration >100 ng/μL confirms sufficient RNA yield.

#### Preparation of linearized DNA templates

16. Before you start, clone, sequence, and purify plasmids encoding the reporter of interest. We use pCRII backbone plasmids encoding a RLuc reporter with a 5′ and a 3′UTR and a template-encoded poly(A) sequence.^5^ To enable co-transcriptional capping a CleanCap-compatible AG initiator after the T7 promoter is necessary. If you opt for enzymatic capping, GGG consensus is preferred for optimal transcription yield. Figure 1 illustrates the features of the plasmid DNA encoding the synthesis of the mRNA reporter.

***Note:*** As an alternative to encoding a poly(A) tail in the DNA template, a separate polyadenylation step can be performed post-transcription using *E.coli* poly(A) polymerase.

**CRITICAL:** When choosing between enzymatic capping (e.g., VCE) and co-transcriptional capping (e.g., CleanCap), consider specific features of the reporter mRNA. For example, short CCC motifs can strongly inhibit translation of enzymatically capped RNAs.^7^

17. Linearize 8 μg plasmid DNA in 200 μL reactions containing the required restriction enzyme sites at the end of the transcript. The point of digestion will signal the end of the transcript during run-off transcription in the next step. As a reference protocol, we set up reactions containing:
  a. 40 ng/μL plasmid.
  b. 1× CutSmart Buffer (NEB).
  c. *Hind*III-HF: Incubate 12–16 h at 37°C using 0.3 U/μL.

***Note:*** To enhance the mRNA stability during translation, linearization should occur immediately after the poly(T) sequence. This can be achieved by using a Type II restriction enzyme such as *Bsm*FI.

***Note:*** The transcription template can be prepared by PCR instead of plasmid linearization.

**Caution:** Select a restriction endonuclease that produces a 5′ overhang or a blunt end of the DNA template. If that is not possible, use Mung Bean Nuclease to blunt the template to avoid dsRNA synthesis.

18. Verify digestion by loading 500 ng DNA on a 1% agarose gel. Run a side-by-side comparison with the same amount of uncut plasmid DNA as a control.

**CRITICAL:** Complete digestion is essential to avoid the production of longer mRNA molecules during *in vitro* transcription.

19. Purify and concentrate the linearized DNA. We suggest using the ChIP DNA Clean & Concentrator Kit (Zymo Research). Elute in 10 μL of elution buffer and use the entire volume for *in vitro* transcription.

### Possible pause point

#### *In vitro* transcription

20. Prepare transcription reactions (100 μL total) with:

**Table:** 

Reagent	Amount	Final concentration	Notes
T7 Transcription buffer (5×)	20.0 μL	1×	Base buffer for transcription
rNTPs (ATP, CTP, GTP, UTP) (100 mM)	1.0 μL each	1 mM each	Final concentration of each nucleotide
Murine RNase Inhibitor (40 U/μL)	2.5 μL	1 U/μL	Protects RNA from degradation due to exogenous contamination
Inorganic Pyrophosphatase (0.1 U/μL)	2.0 μL	0.002 U/μL	Prevents pyrophosphate buildup
T7 RNA Polymerase (20 U/μL)	7.5 μL	1.5 U/μL	Used for runoff transcription
CleanCap AG (100 mM) (optional)	0.8 μL	0.8 mM	For co-transcriptional capping (omit if not used)
Linearized Plasmid DNA	10 μL (7.5 μg)	75 ng/μL	Entire elution volume from prior digestion and purification step
Nuclease-free H_2_O	To 100 μL	–	–

***Note:*** Alternative cap analogs such as ARCA or trinucleotide caps can be used in place of CleanCap; however, their efficiency may vary and should be tested.

***Note:*** The *in vitro* transcription reaction can be scaled up, if larger amounts of RNA are needed.

21. Incubate at 37°C for 1 h.
22. After one hour add 1.5 U/μL T7 RNA polymerase.
23. Incubate for an additional hour at 37°C (total incubation time: 2 h).

#### Plasmid degradation and RNA cleanup

24. Add 0.15 U/μL Turbo DNase (8 μL) to the transcription reaction.
25. Incubate at 37°C for 30 min to degrade template DNA.

**CRITICAL:** Complete DNA digestion is essential to ensure accurate RNA quantification.

26. Purify RNA by using an mRNA purification kit. We routinely use the Monarch RNA Cleanup Kit (NEB). Elute in 20 – 100 μL of 1 mM sodium citrate, pH 6.5.

**CRITICAL:** RNA should always be kept at neutral or slightly acidic pH under RNase-free conditions to avoid alkaline or enzymatic hydrolysis.

#### Post-transcriptional capping

27. Measure RNA concentration via A_260_ spectrophotometry.
28. If you do not opt for co-transcriptional capping, use the Vaccinia Capping System (NEB) following the manufacturer’s instructions. Supplement the reaction with 1 U/μL Murine RNase inhibitor.
29. Purify and quantify the capped RNA as described in step 7.

#### Quality control and storage

30. Quantify mRNAs using Nanodrop and share in RNase-free tubes. Ideal samples exhibit A_260_/A_280_ ratios of ∼2.0 and A_260_/A_230_ ratios of at least 2.0, indicating minimal protein or salt contamination. RNA concentrations above 100 ng/μL were considered sufficient for downstream applications. Expect yields between 50 μg and 400 μg per 100 μL reaction.
31. Incubate 0.5–1 μg of purified mRNA in a formamide containing loading buffer (e.g. NorthernMax, Invitrogen) at 65°C for 5 min and run on a 1% or 2% agarose gel. Single, sharp bands are indicative of high mRNA reporter purity and integrity (Figure 2).

***Note:*** Alternatively use automated electrophoresis systems (like Bioanalyzer or TapeStation) to determine RNA purity and integrity.

**Caution:** Thermal denaturation at 65°C is important to achieve canonical electrophoretic mobility of the mRNA.

32. Snap-freeze in liquid nitrogen and store at –70°C.

**Caution:** Avoid repeated freeze–thaw cycles to preserve RNA integrity.

### Cell-free translation and luciferase assay

**Timing: 2–3 h**

This section describes the procedure for translating *in vitro* transcribed and capped mRNAs using human HeLa S3 lysates. Luciferase-based detection is used to read out translation activity. Alternative readouts, like Western blotting, can be used instead.^6^

#### Reaction assembly and RNA denaturation

33. Thaw lysates and keep all components on ice.
34. Heat the *in vitro* transcribed and capped RNA at 65°C for 5 min in a thermal block. For RNAs with structured 5′UTRs: cool down the tube to 20°C–24°C during 5 min to allow refolding. For unstructured RNAs: cool immediately on ice.
35. Assemble 12.5 μL total reaction volume in RNase-free 1.5 mL tubes as follows:
  a. HeLa S3 lysate to a final concentration of 1 × 10^8^ cell equivalents/mL (stock typically at 2 × 10^8^ cell eq./mL).
  b. Reporter RNA to a final concentration of 5 fmol/μL.
  c. 1 U/μL Murine RNase inhibitor.
  d. Nuclease-free water to volume.
  e. As a negative control reaction, include 0.1 mM cycloheximide (CHX) to inhibit translation.

***Note:*** Consider titrating the reporter mRNA concentration according to the goal of the cell-free translation assay. It is possible to upscale or downscale (minimum of 5 μL) the reaction accordingly. The final concentration of 5 fmol/μL reporter RNA is calculated based on RNA length and mass using the formula: fmol = (mass in ng × 10^6^) / ((RNA length in nt × 320 g/mol) + 18 g/mol).

### Translation incubation

36. Incubate reactions for 50–60 min at 37°C without agitation.
37. After incubation, place all reactions on ice until measuring.

***Note:*** For mechanistic studies, consider performing the reactions at lower temperatures (down to 33°C) if you aim to enrich transient intermediates.

**Caution:** If the assay assesses the impact of recombinant proteins, ensure that negative controls (mutant proteins) are expressed and purified in parallel to ensure that any effects do not relate to co-purified contaminants that affect mRNA stability (nucleases) or protein synthesis.

**Caution:** Different lysates may exhibit variable intrinsic translation efficiencies. To control for this variability, use the same standardized reporter mRNA across lysates as a baseline. Compare the features of the RNA of interest relative to this control to ensure differences reflect RNA-specific effects rather than lysate-dependent variability.

#### Luciferase detection based on Promega Renilla-Glo assay

38. Prepare 1× luciferase detection substrate by diluting 100× stock into luciferase buffer.
39. Add 12.5 μL (or the same adjusted volume as the translation reaction) of the 1× substrate directly to the translation reaction. Mix thoroughly by pipetting up and down 10 times.
40. Transfer the mixture to a white 96-well plate for luminescence measurement. If using 12.5 μL reactions: add 35 μL water to each well for optimal readout. For larger-scale reactions (e.g., 50 μL): transfer directly without dilution.
41. Incubate the plate at 20°C–24°C for 10 min.
42. Measure luminescence using a plate reader (integration time recommended: 0.3 s per well with Promega GloMax Explorer).

***Note:*** Depending on the goal, alternative readouts like Western blotting, polysome gradients etc. can be applied.

**Caution:** Do not compare raw luciferase values across independent experiments, because variations in lysate quality, reagent handling, and instrument settings can affect signal intensity. Always normalize signal intensities to a reference reaction (for example, no treatment with the same reporter mRNA) to enable meaningful comparisons and draw reliable conclusions. On the other hand, while variations between lysate preparations are expected, absolute raw luminescence values obtained from lysates prepared with the same mRNA reporter and measured on the same instrument should remain within a reasonable range (typically within ∼5–10-fold). Larger discrepancies may indicate a batch- or instrument-related problem (e.g., RNA quality, lysate concentration, substrate preparation, or reader calibration) and should be investigated before interpreting fold-change results.

### Measurement of mRNA reporter stability using northern blotting

**Timing: 4 days**

**Figure 3:**
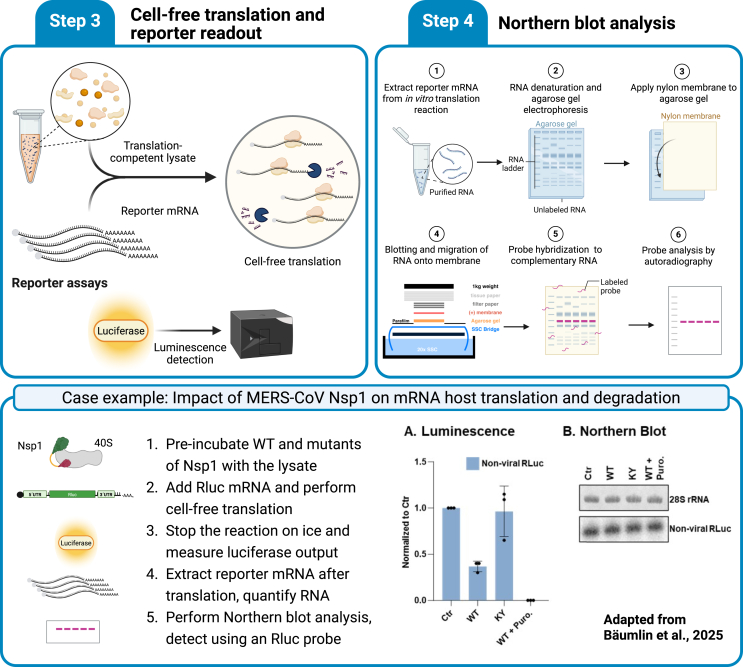
Workflow and example application of the protocol for monitoring mRNA translation and degradation in a human cell-free system (Step 3) Schematic of cell-free translation workflow. Translation-competent HeLa S3 lysates are incubated with reporter mRNAs, followed by measurement of luciferase activity as a readout of translation efficiency. (Step 4) Workflow of Northern blotting to monitor mRNA stability. RNA is extracted from translation reactions, denatured, and separated via agarose gel electrophoresis. RNA is then transferred to a nylon membrane, hybridized with a labeled probe, and detected by autoradiography. Case example: MERS-CoV Nsp1 impairs host mRNA translation without causing reporter mRNA degradation. The translation reaction is supplemented with wild-type (WT) or ribosome-binding mutant (KY) Nsp1 proteins and incubated with an RLuc reporter mRNA. Luminescence measurement (Panel A) shows Nsp1-dependent inhibition of translation. Northern blotting (Panel B) shows no degradation of the reporter mRNA in the presence of WT Nsp1. Data adapted from Bäumlin et al., 2025.^2^

This protocol describes the detection of *in vitro* translated RNAs by Northern blotting, including RNA denaturation, electrophoresis, transfer, probe generation, labeling, hybridization, washing, and signal detection using a phosphor screen. The steps of this part are outlined in Figure 3.

#### RNA isolation

For RNA isolation after *in vitro* translation, phenol-chloroform extraction is compatible with downstream Northern blotting, similar to procedures used for cell-derived RNA.^8^ In our work, we used the Maxwell RSC automated RNA isolation system (Promega). For a detailed analysis of Northern blotting methods, it is advisable to refer to established protocols.^8^

#### RNA denaturation and gel electrophoresis

43. Prepare a 1.2% agarose-formaldehyde gel in 1× MOPS buffer, containing 1% formaldehyde (w/v). Mix 1 part RNA sample (0.5 μg total RNA) with 3 parts NorthernMax Formaldehyde loading dye containing 10 μg/mL ethidium bromide.
44. Denature the RNA at 65°C for 15 min and cool immediately on ice for at least one minute before loading the gel.
45. Load the sample onto the agarose gel.
46. Run the gel at 75 V for 3.5 h or until the dye front reaches 75% of the gel length at 20°C–24°C in 1× MOPS buffer.

**Caution:** For safety, formaldehyde-containing gels should be prepared and run in a fume hood to minimize exposure to toxic fumes.

47. Wash the gels 4 times briefly with MilliQ H_2_O (to remove the excess of formaldehyde).
48. Wash the gel for 7 min in 0.01 N NaOH, 3M NaCl for alkaline hydrolysis.

***Note:*** Alkaline hydrolysis increases RNA mobility during transfer by partially fragmenting the RNA, facilitating more efficient transfer out of the gel. However, hydrolysis should not be excessive, as intact regions are needed for effective probe binding during hybridization.

49. Wash gel briefly twice with water
50. Wash gel briefly with 20× SSC. Keep the gel in 20× SSC until transfer starts.

**CRITICAL:** A prolonged electrophoresis duration ensures the resolution of full-length and partially degraded RNA fragments.

**Caution:** When washing agarose gels, avoid vigorous shaking, especially for gels ≥1.2% agarose, as they are more brittle and prone to breaking. If rocking is required, use only gentle motion and support the gel on a tray when transferring between solutions.

#### Capillary transfer and crosslinking

Transfer RNA from the gel to a positively charged nylon membrane using capillary blotting with 20× SSC buffer 12–16 h at 20°C–24°C.

51. Prewet the membrane briefly by slowly submerging it first in water and then in 20× SSC.
52. Set up the transfer assembly starting from below (see Figure 3, step 4.4):
  a. Buffer chamber filled with 20× SSC.
  b. Thick glass plate.
  c. 2 long Whatman 3MM paper sheets functioning as a 20× SSC bridge.
  d. Agarose gel, directly from hydrolysis.
  e. Parafilm seal around the gel allowing buffer transfer only through the gel.
  f. Positively charged nylon transfer membrane. Cut the top left corner of the membrane (which will be the top right after the transfer) to monitor sample orientation.
  g. 3 Whatman 3MM paper sheets, pre-equilibrated in 20× SSC
  h. 10 cm of tissue paper functioning as capillary force.
  i. 1 kg weight flattening the stack, for example a book.
53. Perform transfer 12–16 h.

**CRITICAL:** To achieve continuous capillary forces, smoothen the surfaces of the membrane and the stacked filter papers after every step with a 50 mL pipette to avoid air bubbles.

54. After transferring, crosslink RNA to the membrane using a UV chamber (Stratalinker), applying 0.120 J during a 1 min exposure.
55. Stain the membrane by incubating it in 0.03% methylene blue prepared in 300 mM sodium acetate (pH 5.5) for 5–10 min at 20°C–24°C with gentle shaking. After staining, rinse the membrane 3–4 times with Milli-Q water until the background is clear. Air dry the membrane face-up on filter paper at 20°C–24°C in the dark to preserve band contrast.
56. Take a digital image of the methylene blue-stained membrane using a flatbed scanner or gel documentation system. This image can be used to normalize for RNA loading and transfer efficiency based on the size and intensity of the 18S and 28S rRNA bands.

### Possible pause

#### Probe synthesis and purification

57. Prepare DNA probes specific to the target RNA by PCR following the guidelines:
  a. Design primers to amplify a region of 200–500 bp, ideally within the open reading frame or 3′UTR of the target RNA. Avoid regions with strong secondary structure or extreme GC content.
  b. Use 0.04 ng/μL plasmid DNA as template.
  c. Use 0.3 μM of each gene-specific primer.
  d. Perform PCR using 1× CloneAmp HiFi Premix (Takara) or an equivalent DNA polymerase, following the manufacturer’s recommended cycling conditions.
58. Gel purification of PCR product:
  a. Add 6× Gel Loading Dye Purple and run the PCR products on a 2% agarose gel with 10 μg/mL ethidium bromide in 1× TAE buffer at 100 V for 20 min.
  b. Visualize DNA under UV and excise the specific band corresponding to the expected probe size.
  c. Excise bands and purify DNA using the NucleoSpin Gel and PCR Clean-up Kit (Macherey-Nagel) according to the manufacturer’s protocol
  d. Elute in RNase-free water and quantify using NanoDrop. Store probes at −20°C until use.
59. Prepare α-^32^P-dCTP labeled single-stranded DNA probe using fresh [α-^32^P]dCTP (half-life: 14.3 days), the purified PCR product from step 9 and the DecaLabel DNA Labeling Kit (Thermo Fisher). We routinely prepare 100 ng of labeled probe per membrane in 50 μL reactions.

***Note:*** Non-radioactive alternatives such as fluorescently labeled probes may also be used for hybridization-based detection, although we have not tested these in this protocol and their efficiency should be validated independently.

60. Purify the labeled probe using MicroSpin G-50 columns (Amersham, Cytiva) following manufacturer’s instructions to remove unincorporated nucleotides. Proceed immediately to hybridization or store short-term at −20°C in a lead-shielded container.

**Caution:** Radiation safety: α-^32^P is a high-energy β-emitter. All procedures involving radioactive material must be conducted in designated radiation areas, using appropriate shielding (e.g., plexiglass), dosimeters, personal protective equipment (PPE), and waste disposal protocols in compliance with institutional regulations.

**CRITICAL:** Probe quality impacts sensitivity: The incomplete removal of unincorporated α-^32^P-dCTP can result in a high background during hybridization. Ensure that the chromatography columns are properly equilibrated and not overloaded to maximize separation efficiency.

**CRITICAL:** Use freshly prepared probes: Radiolabeled DNA degrades over time. For optimal signals, use the labeled probe immediately after purification. If storage is necessary, keep the probe at −20°C in a lead-shielded container and preferentially dilute it in hybridization buffer within 24–48 h.

#### Hybridization and washing

61. Prehybridize the dried membrane in ULTRAhyb Ultrasensitive Hybridization Buffer (Thermo Fisher) preheated to 42°C for at least 30 min.
62. Add the labeled probe to the hybridization buffer to a final concentration of 10 ng/mL.

***Note:*** Use 5–10 mL of hybridization buffer per membrane. Adjust the amount of probe accordingly to achieve the correct concentration. The hybridization buffer containing the diluted probe can be used multiple times within 2–3 weeks; save it after usage or dispose it according to the safety guidelines for the disposal of radioactive material.

**Caution:** Excessive probe may increase background, while insufficient probe amount may reduce sensitivity.

63. Hybridize the membrane 12–16 h at 42°C.

**CRITICAL:** Ensure the membrane is fully submerged in hybridization buffer and incubated in a sealed hybridization tube or bag with gentle rotation to maintain uniform probe distribution.

64. Wash the membrane sequentially to remove nonspecific signals:
  a. Twice with 2× SSC, 0.1% SDS for 5 min each at 42°C.
  b. Twice with 0.1× SSC, 0.1% SDS for 15 min each at 42°C.
65. Seal the moist membrane in a sealable plastic pouch using a heat sealer.

***Note:*** Ensure the membrane remains fully hydrated and flat during sealing to prevent drying or folding, which can affect signal quality.

#### Detection

66. Expose the sealed membrane to a phosphor storage screen 12–16 h in a cassette protected from light.

***Note:*** Ensure the membrane is completely sealed in a bag free of residual wash buffer to avoid smearing or uneven background on the screen.

67. Visualize using a compatible scanner. In our work, we used the Typhoon FLA 9500 scanner (GE Healthcare) with the following settings:
  a. PMT voltage: 1000 V.
  b. Resolution: 100 μm.

**Caution:** Always handle and dispose of radioactive materials (e.g., membranes, screens, gloves) following institutional and national radiation safety regulations.

***Note:*** Signal strength may vary depending on probe incorporation efficiency and membrane exposure time. Optimize scanning sensitivity accordingly.

***Note:*** As an alternative to Northern blotting, mRNA levels can be quantified by reverse transcription followed by quantitative PCR (RT-qPCR). Starting from purified total RNA, dilute the samples to a defined concentration (e.g., 100 ng/μL) in nuclease-free water.

For reverse transcription, typically use 500 ng of total RNA per reaction. The reaction mixture includes random hexamers, reverse transcription buffer, dNTPs, DTT, RNase inhibitor, and a high-fidelity reverse transcriptase. Incubate RNA first with random hexamers at 65°C for 5 min, then cool to 20°C–24°C before the reverse transcriptase is added. Include a parallel control lacking reverse transcriptase to confirm the absence of genomic DNA contamination. Carry out reverse transcription at 50°C for 1 h, and inactivate the enzyme at 75°C for 15 min. Dilute the resulting cDNA (e.g., to 8 ng/μL) for use in qPCR.

Perform quantitative PCR using a SYBR Green–based qPCR master mix and gene-specific primers. Each reaction typically contains 1.6 ng/μL of cDNA and 0.5 μM of each primer. Perform amplification on a real-time PCR system with fluorescence detection. Determine the CT values using standard software setting a manual set threshold. Calculate relative mRNA levels using the comparative CT (ΔΔCT) method, using a housekeeping gene such as GAPDH as an internal normalization control.

## Expected outcomes

The protocol is expected to allow comprehensive studies on mRNA translation and stability, providing reliable and reproducible data for researchers investigating post-transcriptional gene regulation. Based on previous research,^1^^,^^2^^,^^3^^,^^4^^,^^9^ the expected outcomes include the successful preparation of translation-competent HeLa S3 lysates, efficient *in vitro* transcription and capping of reporter mRNAs, robust cell-free translation yielding measurable luciferase activity, and the detection of mRNA stability through Northern blotting.

### Preparation of translation-competent HeLa S3 lysates

The protocol is designed to yield HeLa S3 lysates with high translational activity. By ensuring cell viability above 90% and maintaining cold conditions during preparation, the lysates are expected to retain activity even after multiple freeze-thaw cycles. The final lysate concentration should be approximately 2 × 10^8^ cells/mL to allow downstream *in vitro* translation assays.

### *In vitro* transcription and capping of reporter mRNAs

Using linearized plasmids with appropriate restriction sites, the *in vitro* transcription reactions are anticipated to produce high-quality, capped mRNAs. Co-transcriptional capping with CleanCap AG or enzymatic capping using the Vaccinia Capping System is expected to result in mRNAs with 5′ cap structures, essential for efficient translation initiation. The purified mRNAs are expected to be intact, and thermal denaturation and renaturation should be performed if mRNAs with structured functional elements are assessed.

### Cell-free translation and luciferase assay

When the synthesized mRNAs are introduced into the prepared lysates, robust translation is expected, leading to the production of functional reporter protein. The luciferase activity, measured using a luminescence assay, should correlate with the amount of translated protein in the cell-free system.^6^ The inclusion of controls, such as reactions with cycloheximide or reactions lacking the reporter mRNA, will validate the specificity of translation-dependent signals. The assay is sensitive enough to detect variations in translation efficiency due to different mRNA structures or modifications. If the assay is used to assess the impact of recombinant proteins, ensure that negative controls (mutant proteins) are expressed and purified in parallel to ensure that any effects do not relate to co-purified contaminants that affect mRNA stability (nucleases) or protein synthesis. The protein elution buffer should also be tested to ensure translation compatibility.

### Northern blotting for mRNA stability

The Northern blotting should enable the detection of full-length and degraded mRNA species. By hybridizing radiolabeled probes specific to the reporter mRNAs, researchers can assess the stability and degradation patterns of the transcripts over time. The presence of distinct bands corresponding to intact and fragmented mRNAs will provide insights into the mRNA decay mechanisms under various experimental conditions.

## Quantification and statistical analysis

Measure luminescence values from RLuc assays using a plate reader with an integration time of 0.3 seconds per well. For each experimental condition, at least three biological and three technical replicates were analyzed per experiment. Normalize raw values to a designated control condition (e.g., untreated lysate or control mRNA) within the same experiment to account for batch-specific variability. Process data using Excel or GraphPad Prism, and results are shown as mean ± standard deviation (SD). Perform statistical comparisons between groups using unpaired two-tailed Student’s t-tests or one-way ANOVA as appropriate, with a significance threshold of *p* < 0.05.

Quantify Northern blotting signals from phosphorimager scans (e.g., Typhoon FLA 9500) using ImageJ. Band intensities corresponding to full-length mRNA were normalized to loading controls (e.g., 28S rRNA) or total RNA signal. We quantify at least three independent biological replicates.

## Limitations

While this protocol enables controlled and reproducible analysis of mRNA translation and degradation, it does not fully recapitulate the complexity of living cells. Regulatory features, such as signaling pathways, ribosome quality control, and post-translational modifications, may be absent or only partially active. Translation efficiencies can vary between lysate batches, and the integrity of the RNA is critical for reliable output. Validation in cells is therefore recommended, and the inclusion of a positive control that reflects the mechanism of interest is essential for the interpretation of new results. This protocol is optimized for HeLa S3-derived translation-competent lysates and may not be directly transferable to lysates from other human or mammalian cell lines without further optimization. RNA degradation kinetics can be influenced by the amount of RNA input and the residual endogenous RNA concentration, which may vary across lysate batches. Using radiolabeled probes for Northern blotting also limits throughput and requires appropriate safety infrastructure.

## Troubleshooting

### Problem 1

Low yield or activity of translation-competent lysate (related to lysate preparation steps 1–15). Poor cell viability, uneven lysis, or inappropriate handling conditions can result in suboptimal lysate quality, which directly impacts the efficiency of downstream translation reactions.

### Potential solution

• Ensure that HeLa S3 cells are harvested at the correct density (∼0.8–1.5 × 10^6^ cells/mL) and with >90% viability. Cell health has a direct impact on lysate quality.
• Perform accurate cell counting after brief vortexing to minimize clumping. Repeat counting if values are inconsistent.
• Use pre-cooled buffers and maintain cold temperatures throughout the procedure. Snap-freeze aliquots immediately after preparation and avoid excessive (more than 10) freeze-thaw cycles.
• Avoid excessive pipetting.
• For guidance on producing translation-competent lysate from other cell types, including adherent cells, please refer to Ziegelmüller et al., 2025.^6^

### Problem 2

Uneven or incomplete cell lysis following dual centrifugation (related to lysate preparation steps 1–15). Incomplete lysis compromises yield and reduces translational activity. Dual centrifugation requires precise balance and uniform force across tubes.

### Potential solution

• Distribute equal volumes, ranging from 400 to 800 μL, of gently-mixed cell suspension into dual centrifuge tubes to ensure equal treatment.
• Pre-cool the rotor and ensure the centrifuge reaches the specified temperature (−5°C).
• To assess cell disruption after dual centrifugation, visually inspect the cells using Trypan Blue and adjust the duration or speed accordingly.

### Problem 3

RNA degradation during or after *in vitro* transcription (related to *in vitro* transcription steps 16–32) can result from RNase contamination or improper buffer conditions and compromise downstream translation.

### Potential solution

• Use RNase-free plasticware, certified reagents, and filter pipette tips at all times.
• Elute and store RNA in a mildly acidic buffer (e.g., 1 mM sodium citrate pH 6.4) and keep samples on ice or at −70°C.
• Avoid delays between transcription, DNase treatment, and RNA purification. Do not expose RNA to alkaline pH (>7.5), which promotes hydrolysis.

### Problem 4

Weak or inconsistent luciferase signal in translation assays (related to Cell-Free Translation steps 33–42). A low signal may reflect issues with RNA folding, concentration, capping, poly (A) tail presence, or lysate quality.

### Potential solution

• Heat-denature RNAs at 65°C for 5 min and cool appropriately based on UTR structure. If a structured UTR is required, incubate at 20°C–24°C for 5 min before translation. This is particularly important when assessing the features of viral RNAs that rely their translation efficiency on highly structured sequence elements.^10^^,^^11^^,^^12^ If the UTR should remain unstructured, move RNAs directly on ice.
• Ensure that any additives introduced into the *in vitro* translation reaction do not interfere with translation efficiency or RNA stability. Components such as magnesium, potassium, or trace nuclease contamination may severely inhibit translation or promote mRNA degradation. Ensure that your factor of interest (purified proteins, small RNAs, small molecules) are properly buffered and always verify compatibility with cell-free systems when testing new additives.
• Verify final mRNA concentration that leads to robust translation. Consider testing a dilution range to yield robust luciferase signal (5–80 fmol/μL).
• Include a known positive control RNA to confirm translation efficiency of the lysate.
• Confirm that the luciferase substrate was freshly diluted and uniformly added.

### Problem 5

High background or smearing in Northern blotting related to steps 43–67. Poor resolution or background noise interferes with signal detection and quantification of mRNA stability.

### Potential solution

• Prepare fresh formaldehyde-containing gels and MOPS buffer to ensure appropriate RNA denaturation and separation.
• Run gels long enough to separate full-length from degraded products. Use pre-warmed ULTRAhyb buffer and verify probe quality (by including and probing a reaction lacking the reporter mRNA for potential background) and labeling efficiency (by measuring the efficient incorporation of the radiolabeled nucleotide).
• Increase washing stringency gradually by reducing the salt concentration if high background persists, and monitor membrane exposure time closely.^8^

## Resource availability

### Lead contact

Further information and requests for resources and reagents should be directed to and will be fulfilled by the lead contact, Evangelos D. Karousis (evangelos.karousis@unibe.ch).

### Technical contact

Technical questions on executing this protocol should be directed to and will be answered by the technical contact, Evangelos D. Karousis (evangelos.karousis@unibe.ch).

### Materials availability

This study did not generate new unique reagents.

### Data and code availability

This study did not generate/analyze datasets/code.

## Acknowledgments

This work was supported by grants awarded to E.D.K. from the Swiss National Science Foundation (SNSF; CRSK-3_220624), the Multidisciplinary Center of Infectious Diseases from the University of Bern (MCID), the Fondation Claude et Giuliana, the Forschungsstiftung of the University of Bern, and the Holcim Stiftung. Figures were made using BioRender. We thank Nikolaos Kouvelas, Wojciech Teodorowicz, and Jana Ziegelmüller for critically reading the manuscript.

## Author contributions

E.D.K. and N.S. wrote and edited the manuscript.

## Declaration of interests

The authors declare no competing interests.

## Declaration of generative AI and AI-assisted technologies in the writing process

During the preparation of this work, the authors used GPT-4o in order to improve clarity. After using this tool, the authors reviewed and edited the content as needed and take full responsibility for the content of the publication.
